# Viral and bacterial pathogens identification in children hospitalised for severe pneumonia and parapneumonic empyema

**DOI:** 10.15172/pneu.2012.1/228

**Published:** 2012-11-09

**Authors:** Jean-Noel Telles, Nathalie Richard, Yves Gillet, Susanne Hartwig, Stéphane Pouzol, Sandra Dollet, Melina Messaoudi, Elodie Paredes, Christine Ploton, Gerard Lina, Guy Vernet, Daniel Floret, Etienne Javouhey, Gláucia Paranhos-Baccalà

**Affiliations:** 14 Emerging Pathogens Laboratory, Fondation Mérieux, IFR128 BioSciences Lyon-Gerland, Lyon, France; 24 Service de Réanimation Pédiatrique Médico-Chirurgicale, HFME, Groupement Hospitalier Est, Bron, France; 34 Service de Bactériologie, Groupement Hospitalier Est, Bron, France

**Keywords:** Respiratory pathogens, aetiology, Real-time multiplex PCR, *S. pneumoniae* serotyping

## Abstract

Pneumonia is caused by respiratory bacteria and/or viruses. Little is known if co-infections are an aggravating factor in hospitalised children with severe pneumonia. We studied the impact of respiratory pathogens on the severity of pneumonia. Between 2007 and 2009, 52 children hospitalised with a well-documented diagnosis of community-acquired pneumonia (CAP), with or without parapneumonic empyema (PPE), were enrolled in the study. The patients were classified into 2 groups: CAP + PPE (*n* = 28) and CAP (*n* = 24). The identification of respiratory viruses and bacteria in nasopharyngeal aspirates and pleural effusion samples were performed using conventional bacterial techniques and molecular assays. Using real-time multiplex PCR and antigen detection, *Streptococcus pneumoniae* was the main agent identified in 76% of the cases by molecular tests and BinaxNOW® in pleural fluid. A total of 8% of pleural fluid samples remained undiagnosed. In nasopharyngeal aspirates, rhinovirus, parainfluenza viruses, human metapneumovirus, and respiratory syncytial virus were detected in both CAP and CAP + PPE populations; however, the percentage of viral co-detection was significantly higher in nasopharyngeal aspirates from CAP + PPE patients (35%) compared with CAP patients (5%). In conclusion, viral co-detection was observed mainly in patients with more severe pneumonia. Molecular biology assays improved the pathogens detection in pneumonia and confirmed the *S. pneumoniae* detection by BinaxNOW® in pleural effusion samples. Interestingly, the main *S. pneumoniae* serotypes found in PPE are not the ones targeted by the heptavalent pneumococcal conjugate vaccine.

## 1. Introduction

Community-acquired pneumonia (CAP) remains one of the major causes of mortality in children, especially among those aged less than 5 years. Almost 2 million children died from acute respiratory infections in the year 2000, most from pneumonia [1]. This is more significant in developing countries where incidence rates are up to 10 times greater than in developed countries [1]. In North America, the annual incidence of pneumonia varies from 16 to 22 per 1,000 children older than 5 years and from 30 to 45 per 1,000 children younger than 5 years old [2, 3].

Despite its worldwide impact, there are few reliable data on childhood CAP causative organisms. This is mainly due to the difficulty of establishing the causative diagnosis of CAP aetiology. The majority of CAP cases readily respond to appropriate antibiotic therapy and are considered bacterial rather than viral infections. *Streptococcus pneumoniae* is presumed to be the most frequent bacterial pathogen of lower respiratory tract infection (LRTI) in children [4–8].

Several studies have reported an increasing incidence of childhood community-acquired pleural infection or parapneumonic empyema (PPE) in various countries [9–10]. This pathology is associated with high morbidity and frequently requires prolonged hospitalisation and invasive procedures. To date, several reports have shown a reduction in the overall incidence of invasive pneumococcal disease (IPD) after the introduction of the 7-valent pneumococcal polysaccharide conjugate vaccine, PCV-7, and in some studies the rate increase has been associated with an increase of infections caused by non-PCV7 serotypes [11–14]. Changes in the distribution of *S. pneumoniae* serotypes over time may be associated with changes in clinical type of IPD. The association of serotype 1 with PPE was described in the pre-penicillin and prevaccine eras. Currently, studies have shown an emergence of PPE caused by serotype 1 [15–19]. In this study, we better define the aetiology of severe pneumonia and PPE in hospitalised children using molecular assays. We also identify the *S. pneumoniae* serotypes in the positive specimens.

## 2. Material and Methods

### 2.1 Patients

A prospective study of children hospitalised between April 4, 2007 and March 30, 2009 in the emergency unit and pediatric intensive care unit of Hospices Civils de Lyon for CAP with and without PPE, was performed.

The following data were collected: date of birth, gender, vaccines status, underlying conditions, date of infection, previous antibiotic treatment, clinical signs, presenting signs, symptoms and findings on physical examination, chest radiographs, chest ultrasounds, white blood cell (WBC) counts and C-reactive protein level (CRP), treatment of the current episode (antibiotics, oxygen requirement, chest tube drainage), duration of fever and oxygen requirement, duration of hospitalisation and outcome of the disease. The diagnosis of CAP was based on respiratory complaints and fever, difficulty in breathing, pulmonary infiltrates compatible with pneumonia on the chest radiograph at admission, WBC count >20 G/l or neutrophils >10 G/l and CRP level >60 mg/l after 12 hours of fever. The diagnosis of PPE was based on pleural effusion diagnosed by chest radiographs and confirmed by ultrasounds. The paediatric population was classified into 2 groups: Group 1 CAP − 24 patients with no PPE and Group 2 CAP + PPE − 28 patients with PPE. The groups were further subdivided according to age in 3 different subgroups: <24 months, >24 months and <59 months, and >59 months to 14 years.

### 2.2 Samples

Nasopharyngeal aspirates were obtained from each patient within 48 h of admission using disposable mucus extractors. The samples were kept frozen at −70 °C until further study. Pleural effusion samples were collected in 25 out of 28 cases at the time of the chest drain insertion. Routine bacterial cultures and the BinaxNOW® *S. pneumoniae* latex agglutination test (Inverness Medical Innovations, USA) were performed on the 25 pleural effusion samples obtained. At admission, a blood sample for blood culture was collected from some patients according to their clinical status following the “WHO guidelines on drawing blood” [20] procedure.

### 2.3 Respiratory Pathogens Nucleic Acids Detection

Nasopharyngeal aspirates and pleural effusion samples were submitted for nucleic acid extraction using the NucliSENS® easyMAG platform (bioMérieux, France) in combination with the NucliSENS® magnetic extraction reagents (bioMérieux, France) and NucliSENS® lysis buffer (bioMérieux, France). Briefly, 0.2 ml of clinical sample was added to 2 ml of lysis buffer. The mixture was incubated at room temperature for 10 min before the addition of a mix containing magnetic silica. The mixture was then subjected to the NucliSENS® easyMAG platform for nucleic extraction following the off-board protocol according to the manufacturer’s instructions. Nucleic acids were eluted in 50 µl of elution buffer, and immediately subjected to nucleic acid amplification. The amplification and the detection of the respiratory pathogens was performed using the FTD Respiratory Pathogens Plus assay (Fast Track Diagnostics, Luxembourg) in combination with the AgPath-ID One-Step RT-PCR Kit (Ambion, USA) according to the manufacturers’ instructions. The FTD Respiratory Pathogens Plus assay consists of 6 multiplex realtime (RT)-PCR reactions which detects: influenza A and B, coronaviruses (HCoV) NL63, 229E and OC43, parainfluenza 1, 2, 3 and 4, human metapneumovirus (hMPV) A and B, adenovirus, enterovirus, respiratory syncytial virus (RSV) A and B, rhinovirus, parechovirus, bocavirus, *Mycoplasma pneumoniae, S. pneumoniae, Haemophillus influenzae* 3nd *Staphylococcus aureus*. Ten microlitres of nucleic acid extract was added to each multiplex tube of the FTD Respiratory Pathogens Plus assay and amplification/detection was performed in a CFX96 RT-PCR machine (BioRad, USA).

### 2.4 Serotyping of S. pneumoniae directly from samples

The molecular assay for the *S. pneumoniae* typing is an in-house RT-PCR multiplex molecular assay that identifies the 40 main *S. pneumoniae* serotypes directly from clinical samples without isolation or culture methods. Briefly, 5 µl of nucleic acid extract were added to 8 PCR tubes containing primers and probes for RT-PCR multiplex detection of the following *S. pneumoniae* serotypes (3, 6A/B, 19A, 22F, 4, 9V, 12F, 14, 7F, 11A, 23F, 33F, 16F, SG18, 19F, 35B, 8, 15BC, 31, 38, 1, 10A, 34, 35F, 7C, 15A, 17F, 20 and 5). A LytA internal control is added into the multiplex to confirm the presence of *S. pneumoniae*. The RT-PCR amplifications are performed using the iQ Multiplex Powermix Kit (Bio-Rad, USA) in a CFX96 RT-PCR machine (Bio-Rad, USA).

### 2.5 Ethical Approval

This study was approved by all participating institutional review boards and signed informed consent was obtained from all parents. The approval reference number is: DGS2007-0022MS-01.

## 3. Results

### 3.1 Patient characteristics

Fifty-two consecutively hospitalised children with CAP were enrolled in the study. The patients were classified into CAP (24 cases) and CAP + PPE (28 cases). Demographic and clinical characteristics of the patient groups are compared (Table 1).

**Table 1:** Demographic and clinical characteristics of patients with community-acquired pneumonia (CAP) or community-acquired pneumonia with parapneumonic empyema (CAP + PPE)

	CAP		CAP + PPE		*p* value
Characterisics	*n* ^a^	%	*n*	%	
Overall	24	46	28	54	
Gender					
Male	18	75	12	43	0.025
Age					
3–23 months	6	25	6	21	1
24–59 months	12	50	10	36	0.4
5–14 years	6	25	12	43	0.24
Vaccinated with					
PCV-7^b^	8	33	15	54	0.17
Hib	1	88	24	86	1
Therapy before admission					
NSAID^c^	16	67	22	79	0.4
Antibiotic	7	29	15	54	0.09
Median duration of hospitalisation (days)	3		15		< 0.01
Number of patients treated by oxygen therapy	4	17	22	79	< 0.001
Median duration of symptoms before admission (days)	3		7		0.0065

^a^*n* = total number.

^b^PCV-7 = heptavalent pneumococcal conjugate vaccine

^c^NSAID = Non-Steroidal Anti-Inflammatory Drug

There was no significant difference in gender, age at the admission, vaccinated status and therapies before admission such as antibiotic or non-steroidal antiinflammatory drugs (NSAID) used between the two groups. The median time from onset of symptoms to hospitalisation was significantly shorter in the CAP group (3 days) than in the CAP + PPE group (7 days) (*p* < 0.01). All children with PPE required admission to the paediatric intensive care unit and one third of them required surgical intervention. However, the proportion of children with PPE undergoing surgical intervention decreased with the instillation offibrinolytic therapy into the pleural cavity.

### 3.2 Conventional bacterial culture of pleural effusion and blood samples

Using conventional culture, bacterial agents were poorly identified. Only 1 of the 24 CAP patients and 3 of the CAP + PPE patients were confirmed *S. pneumoniae* positive by blood culture. The serotypes identified in these samples included: serotype 1 (*n* = 2 CAP + PPE), serotype 7F (*n* = 1 CAP + PPE) and serotype 5 (*n* = 1 CAP). Twenty five out of 28 pleural effusion samples were successfully collected from patients with PPE. Bacterial culture of the pleural effusion samples was positive in 5 out of 25 CAP + PPE cases (20%) and identified *S. aureus* producing Panton-Valentine leukocidin PVL (*n* = 1), *S. pneumoniae* (*n* = 1) and *S. pyogenes* (*n* = 3). Thus, conventional bacterial culture identified the bacterial agent in 18% (9/49) of samples.

### 3.3 Bacterial and viral detection in pleural effusion samples in CAP + PPE cases

Of the 25 pleural effusion samples collected, 19 were positive for *S. pneumoniae* using the molecular assay; representing 76% of the total analysed samples. These results were confirmed by the BinaxNOW® *S. pneumoniae* latex agglutination test. The 6 samples negative for *S. pneumoniae* were either *S. pyogenes* (*n* = 3), PVL positive *S. aureus* (*n* = 1) or negative (*n* = 2) by culture.

### 3.4 Serotyping of S. pneumoniae from positive pleural effusion samples

The molecular assay for the *S. pneumoniae* serotyping of the 19 available pleural effusions showed the presence of different serotypes: serotype 1 (*n* = 8), serotype 3 (*n* = 6), serotype 7F (*n* = 3), serotype 19A (*n* = 1) and serotype 22F (*n* = 1). Serotype 3 was found twice in co-infection with serotype 1 and 22F. We identified the same serotype in paired nasopharyngeal and pleural effusion samples in 10 out of 19 CAP + PPE cases tested.

### 3.5 Bacterial and viral detection in nasopharyngeal samples in CAP and CAP + PPE cases

Studying nasopharyngeal aspirates by molecular assays, the viral distribution was similar between the CAP + PPE and CAP (Table 2).

**Table 2:** Respiratory pathogens prevalence in nasopharyngeal samples from 52 children hospitalised for community-acquired pneumonia with parapneumonic empyema (CAP + PPE) or community-acquired pneumonia (CAP)

Pathogens	CAP + PPE (n^a^ = 28)	CAP (n = 24)
Rhinovirus	8	10
Bocavirus	3	0
Parainfluenza viruses	4	4
hMPV^b^	4	1
HCoV-NL-63^c^	2	0
RSV^d^	2	2
Influenza A and B	0	2
Adenovirus	1	0
*M. pneumoniae*	0	2
*S. pneumoniae*	15	21
*S. aureus*	2	2
No pathogen detection	5	3
Mono viral detection	6	17
Dual viral detection	8	1

Data are presented as number of samples positive for bacteria and/or viruses

^a^*n* = total number

^b^hMPV = human metapneumovirus

^c^HCoV-NL-63 = coronaviruses

^d^RSV = respiratory syncytial virus

The main agents found in CAP cases were *S. pneumoniae* (88%) and rhinovirus (42%) followed by parainfluenza virus (17%), RSV and influenza A and B (8%, respectively). Bocavirus (11%), HCoV NL-63 (7%) and adenovirus (4%) were only found in CAP + PPE cases. The viral distribution in the whole population studied is not equal according to the age of the hospitalised patients. Indeed, 83% (10/12) of children from 3 to 23 months old were infected by respiratory viruses whereas only 30% (12/40) of children aged more than 24 months old respiratory virus positive (data not shown). The proportion of dual viral detection is significantly greater in CAP + PPE cases (*n* = 8, representing 35%) than in CAP cases (*n* = 1, representing 5%) (*p* = 0.023). The number of mono viral detection in CAP + PPE (*n* = 6) is significantly lower than in CAP (*n* = 17) (*p* = 0.0003).

## 4. Discussion

Our study presents the aetiological agents in association with the clinical data from 52 hospitalised children in France with well-documented diagnosis of CAP and PPE. Furthermore, data is presented regarding viral and bacterial co-infections in severe pneumonia. Our findings confirm that *S. pneumoniae* is the most common bacterial agent identified in CAP and PPE [19, 22]. *S. pneumoniae* was identified in few samples by blood culture (8%) and by pleural effusion culture (4%). The *S. pneumoniae* detection rose to 76% in pleural effusion samples using the FTD Respiratory Pathogen Plus assay molecular multiplex RT and BinaxNOW® *S. pneumoniae* latex agglutination test. Our data also confirms that conventional bacterial cultures are not sensitive enough to identify the aetiological agent of pneumonia. Before admission, 54% (15/28) of the children in the PPE group were given one or more antibiotics. This could explain why pleural effusion samples were negative for bacteria using conventional culture in the CAP + PPE cases, as has been previously observed [17]. A bacterial aetiological agent can usually be identified when using the BinaxNOW® or molecular assays, even in patients treated with antibiotic therapy [24–26].

In both populations, *S. pneumoniae* was also found in nasopharyngeal aspirates (77%) using a molecular assay, despite the fact that 44% of the patients received the PCV-7. Nasopharyngeal carriage of *S. pneumoniae* is common, especially in young children, thus upper airway samples are not appropriate for pneumococcal culture or direct detection [29]. The vaccine impact analysis for infectious disease is an indirect approach to the evaluation of the importance of the disease when the diagnosis is difficult. The PCV-7 may be used as a probe to define the burden of pneumococcal disease and to better characterise the clinical presentation of pneumococcal pneumonia [11–12, 16]. The reduction in the overall incidence of radiological defined pneumonia suggests that about 30% of episodes of pneumonia are due to *S. pneumoniae*. The PCV-7 was licensed in Europe in 2001 and has been shown to be immunogenic in young children with respect to serotypes 4, 6B, 9V, 14, 18C, 19F, 23F. These serotypes account for between 43% and 75% of IPD in children under the age of 18 years in Western Europe [14]. Serotype 1 remains the most common cause of PPE [11–13, 27–29], but serotypes 3 and 19A are emerging [11]. In our study, we found different serotypes not included in PCV7, including: serotype 1 (9 CAP + PPE), serotype 3 (5 CAP + PPE), serotype 7F (3 CAP + PPE) and serotype 19A (1 CAP + PPE) using our *S. pneumoniae* molecular serotyping assay. The same serotype was identified in paired nasopharyngeal and pleural effusion samples in 10 out of 19 CAP + PPE cases. These data are very relevant for epidemiological studies and to better understand the PPE increase frequency despite the PCV-7 introduction. One of the contributing factors could be the selection pressure induced by vaccination on nasopharyngeal carriage. Continued surveillance will provide crucial data to help to determine vaccine efficacy for the general paediatric population. Moreover, it could also explain the vaccine impact on the frequency of infections caused by pneumococcal isolates with antibiotic resistance and / or serotypes not included in vaccine.

NSAIDs use was described as a risk factor for the severity of the infectious diseases, including pneumonia [19]. Low dosages of NSAIDs such as ibuprofen, which are usually given for antipyresis, have been shown to be proinflammatory, encouraging the influx of neutrophils and increasing the levels of cytokines in the lungs [21]. NSAIDs uses have also been implicated as a risk factor for the development of necrotising fasciitis in children with *S. pyogenes* infections [15, 19, 21–22]. In this study, the number of patients receiving NSAIDs was similar between the two populations, indicating that NSAIDs use doesn’t seem to be a risk factor of PPE.

The exact role of each of the respiratory pathogens in the origin of pneumonia remains to be clarified. However, it has been speculated that viruses may induce pneumonia, either directly or by rendering the host more susceptible to bacterial infection [29–30]. IPD exhibits seasonal variations with peak incidence during the winter season in temperate regions of the world. A variety of host and environmental factors have been explored as potential explanations for the seasonality of IPD including viral respiratory tract infections [29–32]. Viral carriage is rare in healthy subjects and detecting a respiratory virus in upper airway sample either by culture, antigen detection or PCR is considered as diagnosis for the aetiology of LRTI. In nasopharyngeal samples, the viral distribution was similar between the 2 population groups, CAP + PPE and CAP. As already described for bronchiolitis, the number of patients infected with respiratory viruses is much higher in infants less than 24 months old [33]. However, in this study the number of dual viral infections is significantly higher in the PPE population which suggests that viral co-infection seems to have an impact on the severity of pneumonia. Other studies [29–32] have analysed the correlation between IPD and circulating respiratory viruses such as influenza. Up to now, the occurrence of viral co-infections in acute LTRI has received little attention. In a previous study, we described the impact of the dual viral infection in infants admitted to a paediatric intensive care unit associated with severe bronchiolitis [33]. The proportion of viral co-infection in children hospitalised with pneumonia is probably underestimated. Physicians should be aware of the potential outcomes associated with viral co-infection and should consider the impact in children with respiratory illness.

Our data cannot support the hypothesis that viral/bacteria co-infection is a risk factor of PPE in children with CAP, however, it does provide further information regarding the aetiological agents involved in severe pneumonia. Additional studies will be needed to better understand the prevalence of the viral dual infection in pneumonia and the risk factors associated with the severity of the disease. It will be also interesting to determine the impact of the pneumococcal conjugate and influenza vaccinations and antibiotic use in reducing the life-threatening complications of CAP.
